# Prospective observational study of handover in a medical ICU

**DOI:** 10.1186/cc12462

**Published:** 2013-03-19

**Authors:** A Mukhopadhyay, B Leong, A Lua, R Aroos, J Wong, N Koh, N Goh, K See, J Phua, Y Kowitlawakul

**Affiliations:** 1 National University Health System, Singapore; 2 National University of Singapore, Singapore; 3 Alice Lee School of Nursing, National University of Singapore, Singapore

## Introduction

Handovers are often associated with poor communication. ICU patients with multiple complex problems are ideal to study naturally occurring handovers. However, few studies have been conducted in the ICU.

## Methods

We conducted questionnaires of physicians and nurses involved and observed handovers in real time of medical ICU patients over 1 month.

## Results

We interviewed 580 of 672 physicians and nurses involved (86.3%) and observed 90 real-time handovers (45 patients, 26.8%) of 168 patients. Mean duration of handover was 391.3 (± 263.6) seconds, 78.5% were face to face and 1.26 (± 1.75) distractions per handover were noted, person-to-person calling being the commonest mode of distraction (46.7%). Nurses received training during induction in significantly higher numbers, covered allied specialties more and reviewed the patients early (all *P *0.05). Perception of the relative importance of different components of the handover varied significantly between donors, recipients, physicians and nurses. Both physicians and nurses seldom (39.7%) reviewed the available electronic past medical records of the patient before handover, which in addition to training in handover and overall confidence level in the management following handover are significantly associated with better satisfaction in univariate analysis; only the confidence level in patient management remained significant after multivariate analysis. However, agreement between donor and recipient on overall satisfaction was poor (*P >*0.05). Nursing handovers were significantly longer than physicians' (572.08 ± 214.68 vs. 168.6 ± 97.27 seconds, *P *0.001) but are also associated with higher distractions particularly during evening shifts.

## Conclusion

A higher percentage of nurses received handover training; nursing handovers are longer and more inclusive of other components of patient management; perceived importance of components of handover varies among healthcare professionals; distractions are common during handovers and associated with longer duration, by nurses and in the evening shifts; and higher confidence level in patient's management following the handover is associated with better satisfaction.
